# Future impact of thymoquinone-loaded nanoemulsion in rabbits: prospects for enhancing growth, immunity, antioxidant potential and resistance against *Pasteurella multocida*

**DOI:** 10.3389/fvets.2023.1340964

**Published:** 2024-01-16

**Authors:** Marwa I. Abd El-Hamid, Mona M. El-Azzouny, Rania M. S. El-Malt, Mona E. Elkenawy, Abdelwahab A. Abdelwarith, Elsayed M. Younis, Wessam Youssef, Rehab E. Dawod, Dalia W. A. H. Elged, Manal A. M. Habaka, Amal S. A. El Oksh, Soad Mekawy, Simon J. Davies, Doaa Ibrahim

**Affiliations:** ^1^Department of Microbiology, Faculty of Veterinary Medicine, Zagazig University, Zagazig, Egypt; ^2^Department of Bacteriology, Animal Health Research Institute (AHRI), Agriculture Research Center (ARC), Zagazig, Egypt; ^3^Department of Biochemistry, Animal Health Research Institute (AHRI), Agriculture Research Center (ARC), Mansoura, Egypt; ^4^Department of Zoology, College of Science, King Saudi University, Riyadh, Saudi Arabia; ^5^Department of Biotechnology, Animal Health Research Institute (AHRI), Agriculture Research Center (ARC), Giza, Egypt; ^6^Department of Bacteriology, Animal Health Research Institute (AHRI), Agriculture Research Center (ARC), Damietta, Egypt; ^7^Toxicology and Biochemical Department, Animal Health Research Institute (AHRI), Agriculture Research Center (ARC), Zagazig, Egypt; ^8^Department of Poultry and Rabbits Diseases, Animal Health Research Institute (AHRI), Agriculture Research Center (ARC), Zagazig, Egypt; ^9^Department of Biotechnology, Reference Laboratory for Quality Control of Poultry Production (RLQP), Animal Health Research Institute (AHRI), Agriculture Research Center (ARC), Zagazig, Egypt; ^10^Department of Clinical Pathology, Animal Health Research Institute (AHRI), Agriculture Research Center (ARC), Zagazig, Egypt; ^11^Aquaculture Nutrition Research Unit (ANRU), Carna Research Station, College of Science and Engineering, Ryan Institute, University of Galway, Galway, Ireland; ^12^Department of Nutrition and Clinical Nutrition, Faculty of Veterinary Medicine, Zagazig University, Zagazig, Egypt

**Keywords:** thymoquinone nanoemulsions, growth, immunostimulant, antioxidant, anti-virulence, *Pasteurella multocida*

## Abstract

Phytochemical nanoemulsions, such as thymoquinone nanoemulsions (TQN), are regarded as innovative alternatives to antimicrobials that significantly improve the performance, digestion, antioxidant potential and immunity of rabbits. Thus, the potential effects of TQN on growth, digestibility, antioxidant potential, immunity and resistance against *Pasteurella multocida* (*P. multocida*) in rabbits were assessed. Herein, 240 rabbits were offered either a basal diet or diets fortified with three TQN-graded concentrations. At 60 days of age, rabbits were challenged with multidrug-resistant (MDR) virulent *P. multocida* strain. Our outcomes described that dietary inclusion of TQN, especially at higher concentrations, significantly enhanced the growth performance of rabbits, which was supported by increasing the levels of jejunal lipase, amylase and trypsin enzymes. Of note, the levels of muscle and jejunal antioxidant enzymes [superoxide dismutase (SOD), glutathione peroxidase (GPX), catalase (CAT) and total antioxidant capacity (T-AOC)], serum immunological markers (IgG, IgG, IgM and total Igs) and blood phagocytic percentage were significantly provoked after TQN fortification; meanwhile, the levels of muscle and jejunal MDA, serum biochemical parameters (total cholesterol, TG and LDL), abdominal fat percentage, breast and thigh cholesterol were significantly decreased following TQN supplementations. Our findings showed that TQN protected rabbits against *P. multocida* experimental challenge as evidenced by reducing *P. multocida* counts in rabbits’ lungs, downregulating the transcription levels of *P. multocida* virulence-related genes (*ptfA, toxA* and *nanB*) at 48 and 96 h post-infection and ameliorating the expression levels of cytokines-related genes (*IL-1β*, *IL-10, IL-8, IL-6*, *DEFB1*, *TNF-α*, *TLR-4* and *TLR-2*) at 96 h post-infection. Our findings suggest the utilization of TQN in rabbits’ diets due to their stimulating effects on digestibility as well as their growth-promoting, anti-inflammatory, antioxidant, antibacterial, anti-virulence and immunostimulant properties, which enhance the rabbits’ *P. multocida* resistance.

## 1 Introduction

Rabbit’s production is becoming increasingly significant because of its growing role as a major source of animal protein, especially in low-income developing countries (1). Rabbits are highly susceptible to many bacterial diseases severely influencing the rabbit industry and causing substantial economic losses (2). Due to rabbits close contact with other animal species and the environment, they can become carriers of bacterial pathogens (3). Moreover, they are frequently infected with a bacterium called *Pasteurella multocida* (*P. multocida*), which is the common cause of respiratory illness. *P. multocida* is a significant Gram-negative bacterium linked to a variety of animal illnesses. *P. multocida* strains are classified into 16 lipopolysaccharides (LPS) somatic serogroups (1–16) and 5 capsular serotypes (A, B, D, E or F) (4). Some *P. multocida* serogroups can induce snuffles in rabbits (pasteurellosis), which causes severe epidemics and significant financial losses for rabbit breeding worldwide (5, 6). Rabbit pasteurellosis frequently manifests as chronic mucopurulent respiratory distress leading to high mortality rates in rabbits; however, the illness can also be characterized by other clinical manifestations such as septicemia, otitis and abscesses or can develop without exhibiting any clinical indications (7). The consequence of *P. multocida* infections is affected by the complicated interactions of various bacteria-specific and host characteristics (8). The LPS and polysaccharide capsule are significant virulence determinants implicated in the pathogenicity of *P. multocida* (9). Nevertheless, a large number of additional putative virulence factors are connected to the pathogenesis of *P. multocida* such as a wide range of outer membrane proteins, exotoxins, extracellular enzymes, iron acquisition and regulation proteins, fimbriae and colonization and adhesion factors (10). Of note, antimicrobial agents have been utilized for a long time for the treatment and prevention of bacterial infections such as *P. multocida* resulting in the emergence of multidrug-resistant (MDR) pathogens that are known to have a serious threat to the treatment and control of infectious diseases in both animals and humans (11–15). Additionally, the excessive utilization of antimicrobials has serious negative effects including the development of antimicrobial residues in rabbit meat, disturbing the gut microflora and reducing the host defense system (16). As a result, it is critical to find novel alternative antibiotics such as phytochemicals to control bacterial infections brought on by these MDR strains (17, 18).

One of the natural substitutes for antimicrobials is phytochemicals, which are secondary metabolites produced by plants as a result of their interactions with the surrounding environment (19, 20). Phytochemicals such as essential oils (EOs) can be employed as nutritional supplements in animal diets because of their probable positive impacts on health, bacterial loads, immune defense, meat quality, digestion, utilization of nutrients, antioxidant potential and growth performance (19, 21, 22). These beneficial characteristics are related to their function in enhancing mucosal barriers and gut integrity, which augments host immunity and digestion (18, 21, 23). Thymoquinone (TQ) is regarded as the primary phenolic constituent of black cumin (*Nigella sativa;* NS) EO and it has been used for its therapeutic properties in food and animal industries since ancient times (11, 19). Moreover, TQ can be used as a feed additive in animals’ diets owing to its possible beneficial effects on growth performance, utilization of nutrients, immune defense, antioxidant status and microbial load via enhancing digestive and antioxidant enzymes production, gastrointestinal integrity, cytokines-related genes expression and minimizing the harmful bacterial counts (19, 24–26). Nevertheless, the antimicrobial activities of EOs such as TQ might be minimized via volatility, instability issues, low water solubility and oral bioavailability as well as their unpleasant taste (27, 28). To avoid these drawbacks, EOs nanoemulsions such as thymoquinone nanoemulsion (TQN) could be utilized because of their great chemical and physical stability in the aqueous media (28, 29). Due to the EOs’ nanoemulsions nanometric size, they can enhance the EOs’ bioactivity because the nanocarriers can provide precise control over the active substances released at the target location via improving the deep tissue penetration, enhancing the cellular uptake, shielding them from environmental exposure and lowering their volatility (30, 31).

The use of nanotechnology in rabbit breeding remains in its nascent stage. Despite this interest, to the best of our knowledge, there have been no studies on the application of TQN in rabbit breeding. Thus, the present work was undertaken to investigate, for the first time, the *in vivo* effect of TQN on rabbits’ growth performance, digestive and antioxidant enzymes, biochemical and immunological markers in addition to *P. multocida* loads in rabbits’ lung and the expression levels of virulence-and cytokines-related genes post-challenge with virulent *P. multocida* strain.

## 2 Materials and methods

### 2.1 Ethical statement

All procedures for experiments were performed following the rules and authorized specifications of the Institutional Animal Care and Use Committee (IACUC), Faculty of Veterinary Medicine, Zagazig University, Egypt under the reference number ZU-IACUC/2/F/195/2022.

### 2.2 Thymoquinone nanoemulsion preparation and characterization

**Figure 1:**
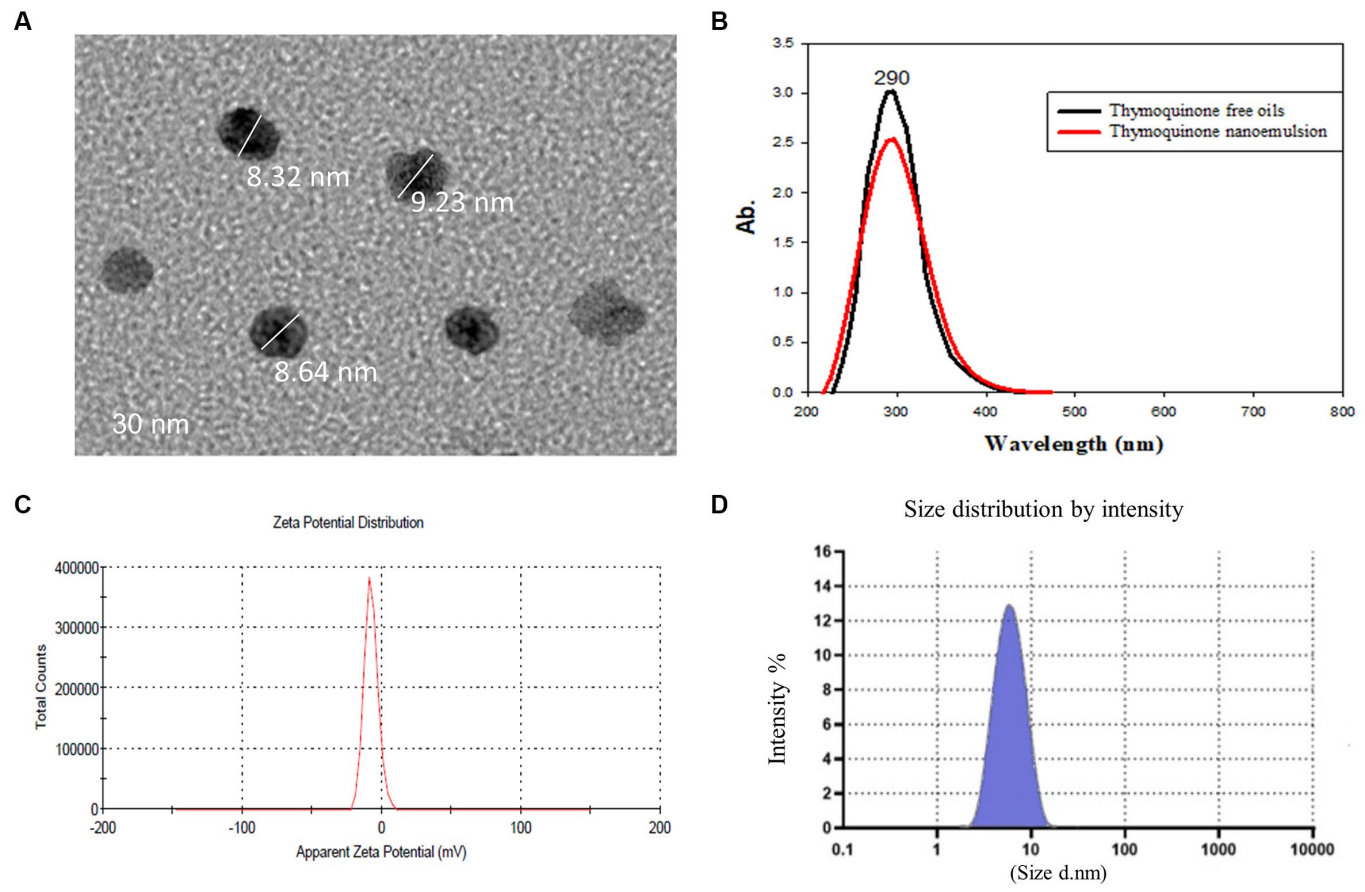
Transmission electron microscopy **(A)**, Fourier transform infrared spectroscopy **(B)**, zeta potential **(C)** and particle size distribution **(D)** of thymoquinone nanoemulsions.

Thymoquinone (03416-100MG, Mol. Wt. 164.20), polyoxyethylene (20) sorbitan monooleate (Tween 80, food grade) and sodium alginate (medium viscosity, A-2033) were obtained from Sigma-Aldrich (St. Louis, MO, United States). In aqueous phase, sodium alginate was completely dispersed in hot water at a temperature of 70°C with continuous stirring until it was completely dissolved. To prepare the oil phase, a lab digital Ultra-Turrax disperser (IKA, Germany) at 3400 rpm for 2 min was utilized and a primary or coarse emulsion was prepared by combining the thymoquinone EO (1% v/v), sodium alginate solution and Tween 80 (1% v/v) as a surfactant. The primary emulsion was then homogenized for 15 min at 10,000 rpm resulting in the creation of the nanoemulsion solution with controlling the temperature via an ice-water cooling jacket to avoid heat build-up during homogenization. A Sonopuls HD 2200 ultrasonicator (Bandelin, Berlin, Germany) was used to sonicate this combination for 10 min at 700 W (31). The average particle size and morphology of synthesized TQN was evaluated via transmission electron microscopy (Figure 1A) at the National Centre for Radiation Research and Technology, Egyptian Atomic Energy Authority, Cairo, Egypt and Fourier transform infrared spectroscopy (Figure 1B) at Radioactive Isotopes and Generators, Atomic Energy Authority, Egypt. Moreover, Zeta potential measurements (Figure 1C) and initial particle size (Figure 1D) were carried out using the dynamic light scattering (Zetasizer Nano ZS, Malvern, UK). The encapsulation efficiency (*EE%*) of TQN was calculated spectrophotometrically via UV-1800 UV–VIS spectrophotometer (Shimadzu, Kyoto, Japan) at 256 nm according to the method described formerly (32) using the following formula: total [(TQN amountis−free TQN amount)/total TQN amount] × 100.

### 2.3 Feeding protocol and experimental design of rabbits

For conducting the present experimental trial, 240 New Zealand weaned male rabbits (30-day-old) were purchased from a local commercial rabbit producer. Once the rabbits arrived, they were weighed separately and then randomly placed into four groups with 60 rabbits and six replicates in each group (10 rabbits/replicate). The four experimental groups consisted of a control group fed the basal diet and three other groups offered meals supplemented with graded levels of TQN including 100, 200 and 300 mg/kg diet, which were spread uniformly over the feed by spraying after the pelleting process during the 30 days feeding trail. The animals were kept in cages under hygienic conditions during the feeding phase and offered pelleted diets with unlimited access to food and water *ad libitum*. The basal diets were created using the nutrient recommendations (33) as displayed in Table 1.

**Table 1:** The control experimental diet’s ingredients and nutrition levels.

	%
**Ingredient**	
Barley grains	16.3
Molasses	3.00
Wheat bran	19
Berseem hay	33.2
Soybean meal, 44%	15.70
Yellow corn	10
Common salt	0.5
Calcium dibasic phosphate	1.5
Anticoccidial	0.2
Antitoxin	0.3
Premix^a^	0.3
**Nutrient composition**	
Digestible energy (KcaL/Kg)	2555.60
Phosphorus (%)	0.59
Calcium (%)	1.09
Crude fiber (%)	12.56
Ether extract (%)	2.33
Crude protein (%)	16.33

a Premix: each 5 kg diet consists of vitamins: B12, 2 mg; B6, 200 mg; B2, 600 mg; B1, 200 mg; E, 3,200 mg; D3, 3,000,000 IU and A, 1800,000 IU; magnesium, 100 g; Zn, 12,000 mg; Se, 20 mg; Co, 20 mg; I, 200 mg; Cu, 3,000 mg; Mn, 10,000 mg; Fe, 10,000 mg; choline, 10,000 mg; nicotinic acid, 4,400 mg and Ca antothenate, 2000 mg.

### 2.4 Growth performance attributes

The initial body weight of rabbits in each group was estimated at the start of the growing period. After that, the rabbits’ body weight and feed intake (FI) were recorded weekly to determine the growth performance aspects during the feeding period (60 days old). The feed conversion ratio (FCR), body weight gain (BWG) and FI were calculated as previously described (11, 34–36).

### 2.5 Collection of samples

After the end of the rearing period (60 days old), blood samples were aseptically collected from ear veins of 24 experimental rabbits (6/group). The blood samples were then separated into two portions; the first one was obtained in sterile centrifuge tubes with an anticoagulant (ethylenediaminetetraacetic acid, Sigma, United States) to be used for determining the phagocytic percentage and hematological parameters and the second portion was obtained in a sterile centrifuge tube without an anticoagulant for separation of sera through centrifugation at 2,000 rpm for 10 min and the obtained sera were kept at −20°C for assessing the immunological and biochemical markers. Additionally, 3 rabbits from each replicate were randomly captured, fasted overnight, weighed and then sacrificed via cervical dislocation according to the guidelines of the World Rabbit Science Association (37) to determine the yields of abdominal fat. After that, the breast muscle and jejunal samples were utilized for determining the activities of digestive and antioxidant enzymes. Moreover, the breast and thigh muscle samples were used to detect the total cholesterol levels in the thigh and breast.

### 2.6 Analysis of the digestive and antioxidant enzymes’ activities

The activities of amylase, lipase and trypsin enzymes were determined in the jejunal samples utilizing commercial kits (Nanjing Jiancheng Bioengineering Institute, Nanjing, China) according to the company’s directions. Additionally, the activities of antioxidant enzymes including superoxide dismutase (SOD), glutathione peroxidase (GPX) and catalase (CAT) as well as the levels of malondialdehyde (MDA) and total antioxidant capacity (T-AOC) in breast muscle and jejunal tissues were determined using commercial kits (Nanjing Jiancheng Bioengineering Institute, Nanjing, China) following the producer’s manuals. The principle of the used commercial kits is based on the reaction between prepared samples and specified chemicals and the colored products were assayed colorometrically.

### 2.7 Biochemical and immunological markers

Red blood cells (RBCs) were assessed by a means of a Neubauer hemocytometer (Sigma, Germany) and hemoglobin (Hb) values were estimated via the cyanomethemoglobin colorimeteric technique. The levels of serum alanine and aspartate aminotransferase (ALT and AST), low-density lipoprotein (LDL), total cholesterol (TC) and total triglycerides (TG) were detected utilizing analytical kits (Spinreact Co., Santa Coloma, Spain) following the procedures’ manuals. The yields of abdominal fat were represented as a proportion of the body weight. Moreover, the breast and thigh muscle samples were used to enzymatically detect the total cholesterol in the thigh and breast via gas chromatography as in agreement with Association of Official Agricultural Chemists (AOAC) International 2002-AOAC 994.10 standard (38). The examined samples were processed chemically via saponification with 5% KOH in methanol, pH = 2, extraction in ether, concentration and suspension in chloroform and they were subsequently analyzed using gas chromatography.

The serum concentrations of immune-related variables including immunoglobulin M (IgM), IgG and IgA were evaluated utilizing the enzyme-linked immunosorbent assay kits (Sigma Aldrich, MO, United States) as per the manufacturer’s regulations. Furthermore, serum total immunoglobulins (Igs) and blood phagocytic percentage were determined as stated previously (39, 40).

### 2.8 *Pasteurella multocida* challenge trial

The MDR virulent *P. multocida* strain used in the current challenge study was isolated from rabbits with respiratory signs of snuffles and phenotypically identified according to conventional microbiological techniques. Briefly, the strain was grown into blood agar medium and the developed colonies were stained with Gram’s stain and then examined microscopically. Definitive identification was further conducted using various biochemical tests comprising oxidase, catalase, methyl red, Voges-Proskauer, indole, citrate, H_2_S production and urease consistent with standard techniques (6, 41). The utilized strain was molecularly identified using PCR examination of a species specific gene fragment; *kmt1* gene as in compliance with the previously pronounced procedure (42). Additionally, the strain was confirmed to be MDR after examining its antimicrobial susceptibility pattern according to the European Committee on Antimicrobial Susceptibility Testing (EUCAST) standard (43). Moreover, it was affirmed to be virulent via PCR testing for the presence of genes encoding dermonecrotoxin (*toxA*), sialidases (*nanB*) and colonization and adhesion-related protein (*ptfA*) utilizing primers and PCR cycling protocols formerly described (6, 44, 45). The infecting strain was enriched in brain heart infusion broth (Oxoid, UK), passed twice in healthy rabbits for improving its pathogenicity and then it was re-isolated from sacrificed rabbits and used for the challenge trial. All rabbits were checked to make sure they were *P. multocida*-free before starting the challenge trial via bacteriological examination of conjunctival, nasal and rectal swabs for isolation and phenotypic and molecular identification of *P. multocida* as previously detailed. At 60 days of age (the end of rearing period), 18 animals representing 6 replicates from each group were challenged intranasally with 0.1 mL of pure *P. multocida* inocula containing 2 × 10^5^ CFU/mL in sterile phosphate buffer saline. The experimentally infected rabbits were monitored for 96 h to look for any emergence of clinical signs. Additionally, clinically diseased, moribund and freshly dead rabbits were aseptically submitted for re-isolation and identification of the infecting *P. multocida* strain.

#### 2.8.1 Quantification of *Pasteurella multocida*

At 48 and 96 h post-infection with *P. multocida*, 6 rabbits per each experimental group were euthanized and colony-forming units (CFUs) of *P. multocida* strains were detected in lung, spleen and liver tissues after culturing onto blood agar medium supplemented with 5% fresh sheep blood and clindamycin.

#### 2.8.2 Gene expression analysis of virulence- and cytokines-related genes utilizing reverse transcription-quantitative PCR technique

Lung and splenic tissues were collected from 6 rabbits per each experimental group and used to extract total RNA using the QIAamp RNeasy Mini kit (Qiagen, Hilden, Germany) according to the producer’s manuals. A Nano Drop 2000 spectrophotometer (Thermo Scientific Inc., Waltham, MA, United States) was used for evaluating the RNA concentration and purity. The gene expression profiling was performed utilizing the 2x QuantiTect SYBR Green RT-PCR Kit (Qiagen, Hilden, Germany) via one-step reverse transcription-quantitative polymerase chain reaction *(*RT-qPCR) tests. The mRNA transcript levels of virulence-related genes (*ptfA, toxA* and *nanB*) were determined at 48 and 96 h post-infection and the expression levels of cytokines-related genes including interleukin-1β (*IL-1β*), *IL-10, IL-8, IL-6*, beta-defensin 1 (*DEFB1*), tumor necrosis factor-alpha (*TNF-α*), toll-like receptor 4 (*TLR-4*) and *TLR-2* were determined at 96 h post-infection. A Stratagene Mx3005P real-time thermal cycler (Agilent Technologies, Inc., Santa Clara, CA, United States) was utilized to perform each RT-qPCR reaction in triplicate. The transcript levels of the housekeeping genes, glyceraldehyde 3-phosphate dehydrogenase (GAPDH) and *kmt1* genes were served as endogenous controls for the expression levels of the examined genes. Table 2 describes all primer sequences of the studied genes. Post-PCR melting curve analyses were used to confirm the specificity of the PCR amplifications and the purity of the qPCR products after completing the RT-qPCR procedures. The 2^−∆∆Ct^ technique was used to analyze the relative modifications in gene transcription levels (46).

**Table 2:** Primers’ sequences of the investigated genes used in reverse transcription-quantitative PCR techniques.

Specificity/target gene	Primer sequence (5′-3′)	Accession No./reference
**House keeping**		
*GAPDH*	F-GGTGGTGCTAAGCGTGTTA	NM205518
	R-CCCTCCACAATGCCAA	
*kmt1*	F-ATCCGCTATTTACCCAGTGG	(42)
	R-GCTGTAAACGAACTCGCCAC	
**Virulence attributes**		
*ptfA*	F-TGTGGAATTCAGCATTTTAGTGTGTC	(45)
	R-TCATGAATTCTTATGCGCAAAATCCTGCTGG	
*toxA*	F-CTTAGATGAGCGACAAGG	
	R-GAATGCCACACCTCTATAG	
*nanB*	F-CATTGCACCTAACACCTCT	
	R-GGACACTGATTGCCCTGAA	
**Cytokines**		
*IL-1β*	F-TTCCGGATGTATCTCGAGCA	NC_013670
	R-GTGGATCGTGGTCGTCTTCA	
*IL-10*	F-AAAAGCTAAAAGCCCCAGGA	NM001082045.1
	R-CGGGAGCTGAGGTATCAGAG	
*IL-8*	F-CTCTCTTGGCAACCTTCCTG	KT216053.1
	R-TTGCACAGTGAGGTCCACTC	
*IL-6*	F-GCCAACCCTACAACAAGA	NC_013678
	R-AGAGCCACAACGACTGAC	
*DEFB1*	F-AGCCTGTCTGCCTGGAGTAG	XM017337690.1
	R-GATGAGGAGAGGCTTCATGG	
*TNF-α*	F-CTGCACTTCAGGGTGATCG	XM_008262537.2
	R-CTACGTGGGCTAGAGGCTTG	
*TLR-4*	F-AGATGAAGTTGTTCCCTCCG	NM_001082732.2
	R-GTGGGCTTAGAACAACTGGAAC	
*TLR-2*	F-TGCCTCCTTGTTACCTATGC	NM_00108271
	R-AGATGAAGTTGTTCCCTCCG	

*GAPDH*, glyceraldehyde 3-phosphate dehydrogenase; *ptfA*, *Pasteurella multocida* type 4 fimbrial subunit gene; *toxA*, dermonecrotoxin gene; *nanB*, gene encoding *P. multocida* sialidase protein; *IL*, interleukin; *DEFB1*, beta-defensin 1; *TNF-α*, tumor necrosis factor-alpha and *TLR*, toll-like receptor.

### 2.9 Statistical analysis

Our data were analyzed using the general linear model of SPSS Inc. program version 20 (IBM Corp., Armonk, NY, United States) and the statistical significance difference between the experimental groups was evaluated using Tukey’s posthoc test. Levene’s and Shapiro–Wilk’s tests were used to determine the homogeneity and normality among the treatment groups, respectively. The results were expressed as the standard error of means (SEM) and the significance was identified at *p* < 0.05. The GraphPad Prism program version 8 (San Diego, CA, United States) was used to create the graphs in the current study.

## 3 Results

### 3.1 Thymoquinone nanoemulsion characterization

The average particle size and zeta potential of synthesized TQN are illustrated in Figure 1, where TQN exhibited a negative average zeta potential of −23 ± 0.89 mV and initial particle size of 8.73 ± 1.20 nm with polydispersity index of 0.022 and EE% of 87.65 ± 0.42%.

### 3.2 Growth performance and activities of digestive enzymes

Table 3 displays the findings of rabbits’ growth performance criteria following dietary inclusion of different levels of thymoquinone nanoemulsions. After ending the rearing period (60 days old), rabbits offered diets with TQN supplementations at the levels of 200 and 300 mg/kg showed prominently significant (*p* < 0.001) rises in BWG (1,613 and 1,463 g, respectively). On the other hand, the BWG of rabbits supplemented with 100 mg/kg of TQN (1,392 g) and those offered a control basal diet (1,378 g) showed no significant variations. Additionally, the best (*p* = 0.03) FCR was seen in rabbits offered diets with TQN supplementation at doses of 200 and 300 mg/kg (2.57 and 2.50, respectively) in comparison with the control group (3.38).

**Table 3:** Impact of different levels of thymoquinone nanoemulsions on rabbits’ growth performance traits and jejunal digestive enzymes’ activities over the whole rearing period (60 days old).

Parameter		Experimental groups				
	Control	TQNI	TQNII	TQNIII	*p* value	SEM
**Growth performance**						
Body weight, g	2190^c^	2205^c^	2269^b^	2419^a^	< 0.001	14.56
Body weight gain, g	1378^c^	1392^c^	1463^b^	1613^a^	< 0.001	11.10
Feed intake, g	4661^a^	4559^a^	3755^b^	4028^c^	0.02	13.30
Feed conversion ratio	3.38^a^	3.28^b^	2.57^c^	2.50^c^	0.03	1.34
**Digestive enzymes**						
Amylase (U/g prot)	254.34^c^	255.88^c^	265.16^b^	299.90^a^	<0.001	1.03
Lipase (U/g prot)	437.05^d^	459.18^c^	479.56^b^	490.02^a^	<0.001	1.47
Trypsin (U/g prot)	280.86^b^	295.79^a^	300.2^a^	302.04^a^	<0.001	1.07

Control, rabbits fed basal control diets; TQNI, TQNII and TQNIII, rabbits fed basal diets with thymoquinone nanoemulsions (TQN) supplementations at graded levels consisting of 100, 200 and 300 mg/kg diets, respectively; SEM, standard error of the mean. ^a-d^ Means in a row with different superscript letters represent statistical variations (*p* < 0.05).

The effect of supplementing graded levels of TQN on the activities of digestive enzymes in the rabbits’ jejunal tissues is shown in Table 3. The results showed that increasing TQN levels significantly (*p* < 0.001) raised the activities of jejunal amylase and lipase enzymes in a dose-dependent manner at 60 days old. Additionally, the most notable (*p* < 0.001) increases in the activities of jejunal amylase, lipase and trypsin enzymes (299.90, 490.02 and 302.04 U/g prot, respectively) were noticed in rabbits administered TQN at a concentration of 300 mg/kg.

### 3.3 Analysis of antioxidant-related parameters and malondialdehyde content

Table 4 describes the impact of dietary inclusion of different levels of TQN on the activities of antioxidant parameters in addition to MDA contents in breast muscle and jejunal tissues of rabbits. Surprisingly, significant improvements (*p* < 0.001) were seen in the activities of T-AOC, SOD, GPX and CAT in the breast muscle and jejunal tissues of rabbits supplemented with higher levels of TQN unlike the control group. Moreover, the most significant (*p* < 0.001) increase in the levels of T-AOC (14.9 U/mg prot), SOD (35.35 μ/mL), GPx (322.32 μmoL/mg) and CAT (5 U/L) were detected in the breast muscles of rabbits fortified with TQN at concentrations of 300 mg/kg in comparison with the control group (8.26 U/mg prot, 31.03 μ/mL, 295.78 μmoL/mg and 3.2 U/L, respectively). Furthermore, the most significant (*p* < 0.001) elevation in the activities of T-AOC (25.8 U/mg prot), SOD (20.16 μ/mL), GPx (302.8 μmoL/mg) and CAT (4.02 U/L) was seen in the jejunal tissues of rabbits fortified with TQN at concentrations of 300 mg/kg in comparison with the control group (17.92 U/mg prot, 17.9 μ/mL, 253.18 μmoL/mg and 2.64 U/L, respectively). On the other hand, muscle and jejunal MDA levels were significantly decreased (*p* < 0.001) across all TQN-supplemented groups as the level of TQN fortification increased. Additionally, the most significant (*p* < 0.001) decreases in the activities of muscle and jejunal MDA (0.2 and 1.28 nmoL/mL, respectively) were determined in rabbits offered TQN at the level of 300 mg/kg concerning the control group (0.38 and 2.06 nmoL/mL, respectively).

**Table 4:** Effect of various levels of thymoquinone nanoemulsions on the activities of antioxidant markers in the breast muscle and jejunal tissues of rabbits.

Antioxidant markers		Experimental groups				
	Control	TQNI	TQNII	TQNIII	*p* value	SEM
**Breast muscle**						
T-AOC (U/mg prot)	8.26^c^	10.04^b^	11.00^b^	14.90^a^	< 0.001	0.25
SOD (μ/mL)	31.03^c^	32.09^b^	34.92^a^	35.35^a^	< 0.001	0.08
GPX (μmoL/mg)	295.78^c^	295.88^c^	319.7^b^	322.32^a^	< 0.001	0.36
CAT (U/L)	3.20^c^	3.48^c^	4.14^b^	5.00^a^	< 0.001	0.08
MDA (nmoL/mL)	0.38^a^	0.29^a^	0.24^b^	0.20^c^	< 0.001	0.01
**Jejunal tissues**						
T-AOC (U/mg prot)	17.92^c^	18.64^c^	21.62^b^	25.80^a^	< 0.001	0.15
SOD (μ/mL)	17.90^c^	18.12^bc^	18.92^b^	20.16^a^	< 0.001	0.12
GPX (μmoL/mg)	253.18^c^	256.12^c^	276.58^b^	302.8^a^	< 0.001	1.5
CAT (U/L)	2.64^c^	3.27^b^	3.72^a^	4.02^a^	< 0.001	0.05
MDA (nmoL/mL)	2.06^a^	1.70 ^ab^	1.48^b^	1.28^c^	< 0.001	0.04

T-AOC, total antioxidant capacity; SOD, superoxide dismutase; GPX, glutathione peroxidase; CAT, catalase; MDA, malondialdehyde; control, rabbits fed basal control diets; TQNI, TQNII and TQNIII, rabbits fed basal diets with thymoquinone nanoemulsions (TQN) supplementations at graded levels consisting of 100, 200 and 300 mg/kg diets, respectively; SEM, standard error of the mean. ^a-c^Mans in a row with different superscript letters represent statistical variations (*p* < 0.05).

### 3.4 Analysis of serum biochemical parameters, abdominal fat and muscle cholesterol

The impact of dietary graded levels of TQN on the rabbits’ serum biochemical parameters, abdominal fat and muscle cholesterol are summarized in Table 5. Notably, there were no significant differences (*p* > 0.05) in the RBCs counts, Hb concentrations and serum levels of AST and ALT among all experimental groups. Moreover, the lipid profile revealed that total cholesterol, TG and LDL levels were decreased in the sera of rabbits with increasing dietary levels of TQN. The most significant (*p* < 0.05) reductions in the serum levels of TC, TG and LDL (108.36, 75.82 and 94.44 mg/ dL, respectively) were detected among rabbits fortified with TQN at a level of 300 mg/Kg when compared with the control group (112.44, 78.9 and 111.5 mg/ dL, respectively).

**Table 5:** Impact of graded levels of thymoquinone nanoemulsions on the levels of hematology, serum biochemical parameters, abdominal fat and muscle cholesterol levels in rabbits.

Parameter		Experimental groups				
	Control	TQNI	TQNII	TQNIII	*p* value	SEM
RBCs (×10^6^/μL)	2.62	2.67	2.69	2.72	0.07	0.10
Hb (g/dL)	11.29	11.27	11.30	11.33	0.80	0.12
WBC, 10^3^/mL	12.55	11.99	12.03	12.13	0.60	0.15
Platelets, 10^3^/mL	125.66	126.87	125.99	126.12	0.73	0.46
ALT (U/L)	21.18	21.36	20.88	21.09	0.575	0.12
AST (U/L)	51.40	51.36	50.54	51.66	0.094	0.152
TG (mg/dL)	78.9^a^	78.58^a^	76.62^b^	75.82^b^	< 0.001	0.188
TC (mg/dL)	121.44^a^	118.84^b^	113.24^c^	108.36^d^	< 0.001	0.322
LDL (mg/dL)	111.50^a^	99.58^b^	98.44^b^	94.44^c^	< 0.001	0.378
Abdominal fat (%)	2.11^a^	2.06^b^	2.05^b^	1.91^b^	0.003	0.017
Breast cholesterol (mg/100 mg)	59.36^a^	59.27^a^	57.43^b^	56.52^b^	< 0.001	0.209
Thigh cholesterol (mg/100 mg)	62.24^a^	61.73^ab^	61.73^ab^	60.52^b^	0.028	0.185

RBCs, red blood cells; Hb, hemoglobin; ALT, alanine aminotransferase; AST, aspartate aminotransferase; TG, total triglycerides; TC, total cholesterol; LDL, low-density lipoprotein; control, rabbits fed basal control diets; TQNI, TQNII and TQNIII, rabbits fed basal diets with thymoquinone nanoemulsions (TQN) supplementations at graded levels consisting of 100, 200 and 300 mg/kg diets, respectively; SEM, standard error of the mean. ^a-d^Means in a row with different superscript letters represent statistical variations (*p* < 0.05).

Remarkably, supplementing rabbits with TQN had a decreasing impact on their abdominal fat percentages. Moreover, the most significant (*p* = 0.003) decline in the abdominal fat was seen in rabbits offered TQN dietary supplementation at a concentration of 300 mg/Kg (1.91%) concerning the control group (2.11%). Compared to the thigh, rabbits’ breast muscle had less cholesterol levels and raising the intake of dietary TQN decreased the cholesterol levels in both tissues concerning the control group. Furthermore, the most significant (*p* < 0.01) minimizations in the concentrations of thigh and breast cholesterol (60.52 and 56.52 mg/100 mg, respectively) were pronounced in rabbits fortified with TQN at the level of 300 mg/Kg regarding the control group (62.24 and 59.36 mg/100 mg, respectively).

### 3.5 Analysis of serum immunological parameters and blood phagocytic percentage

Data relating to the serum immunological parameters and blood phagocytic percentage post supplemnting rabbits with graded TQN levels are displayed in Table 6. Concerning the concentrations of serum IgG, IgM, IgA, total Igs and blood phagocytic percentage, they were significantly (*p* < 0.001) upraised in rabbits offered TQN dietary inclusion in a dose dependant manner. Notably, rabbits offered TQN at a concentration of 300 mg/Kg showed the highest significant (*p* < 0.001) immune response as evinced by increased serum levels of IgG, IgM, IgA, total Igs and blood phagocytic percentage (13.3, 0.69, 0.81 and 16.52 mg/ dL and 49.12%, respectively) when compared with the control group (10.84. 0.48, 0.62 and 12.7 mg/ dL and 25.4%, respectively).

**Table 6:** Impact of different concentrations of thymoquinone nanoemulsions on the levels of serum immunological parameters and blood phagocytic percentage in rabbits.

Parameter		Experimental groups				
	Control	TQNI	TQNII	TQNIII	*p* value	SEM
IgG (mg/dL)	10.84^c^	11.64^b,c^	11.96^b^	13.30^a^	< 0.001	0.142
IgM (mg/dL)	0.48^c^	0.58^b^	0.64^a,b^	0.69^a^	< 0.001	0.11
IgA (mg/dL)	0.62^c^	0.66^c^	0.74^b^	0.81^a^	< 0.001	0.008
Total Igs (mg/dL)	12.70^d^	14.68^c^	15.52^b^	16.52^a^	< 0.001	0.094
Phagocytic percentage (%)	25.40^d^	38.92^c^	42.50^b^	49.12^a^	< 0.001	0.305

Ig, immunoglobulin; Igs, immunoglobulins; control, rabbits fed basal control diets; TQNI, TQNII and TQNIII, rabbits fed basal diets with thymoquinone nanoemulsions (TQN) supplementations at graded levels consisting of 100, 200 and 300 mg/kg diets, respectively; SEM, standard error of the mean. ^a-d^Means in a row with different superscript letters represent statistical variations (*p* < 0.05).

### 3.6 Effect of thymoquinone nanoemulsions dietary inclusion on *Pasteurella multocida* counts

**Figure 2:**
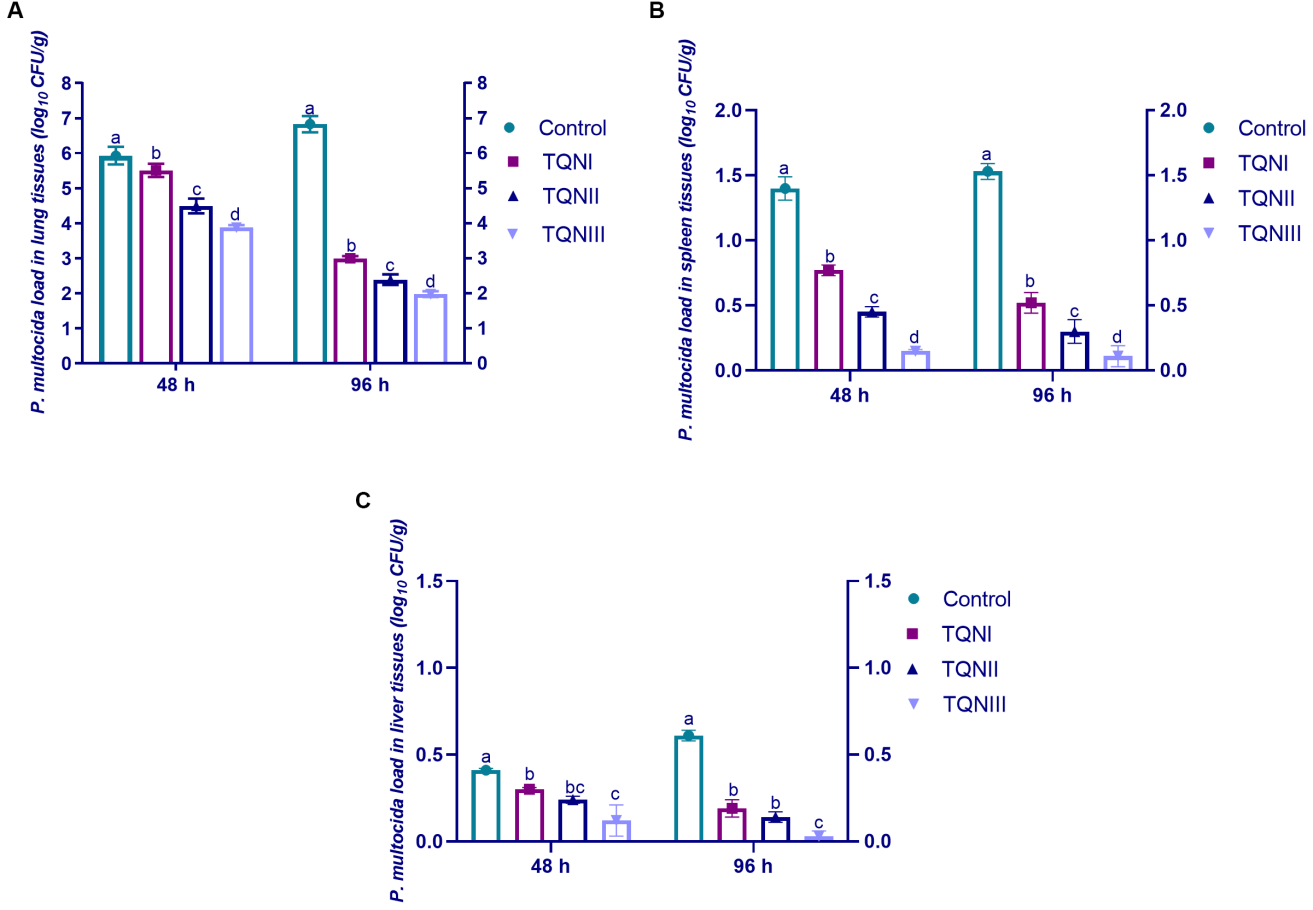
Quantification of *P. multocida* loads in the lung **(A)**, spleen **(B)** and liver **(C)** tissues of rabbits in response to thymoquinone nanoemulsions (TQN) supplementations at 48 and 96 h post-infection with MDR virulent *Pasteurella multocida* strain. Values are means ± standard error of the mean (SEM) in bars. Control, rabbits fed basal control diets; TQNI, TQNII and TQNIII, rabbits received basal diets with TQN supplementations at graded levels consisting of 100, 200 and 300 mg/kg diets, respectively. ^a–d^Means with various superscript letters denote statistical variations (*p* < 0.05).

At 48 and 96 h post-infection with MDR virulent *P. multocida* strain, TQN-supplemented rabbits showed no observable clinical symptoms of snuffles or subcutaneous hemorrhages in contrast to control rabbits, which showed respiratory clinical signs of snuffles, subcutaneous hemorrhages and decline in their activities. The quantification outcomes of *P. multocida* in the lung, spleen and liver tissues of experimentally infected rabbits are demonstrated in Figure 2. At 48 and 96 h post-infection, *P. multocida* was numerically and significantly (*p* < 0.05) reduced in the lung and spleen tissues of rabbits offered TQN supplementations in a dose-dependent manner. Notably, our data showed that *P. multocida* populations were at their lowest levels in lung, spleen and liver tissues of rabbits receiving TQN supplementations at a level of 300 mg/Kg at both time intervals post-infection with *P. multocida* strain (up to 1.89, 0.11 and 0.03 log_10_ CFU/g, respectively).

### 3.7 Gene expression analysis of virulence-related genes post-infection with *Pasteurella multocida*

**Figure 3:**
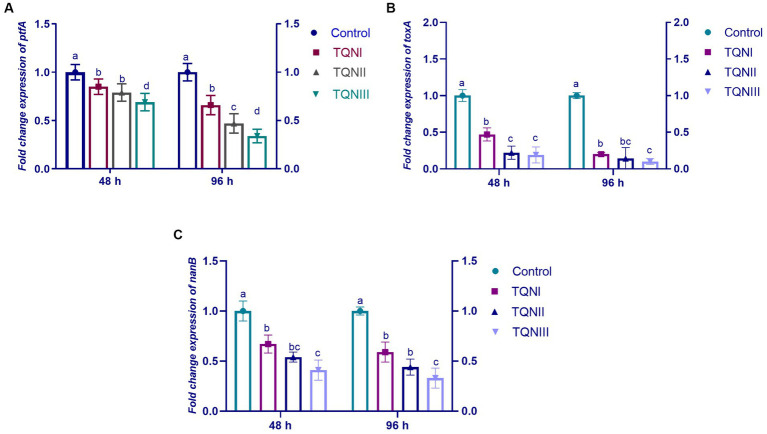
Impact of dietary fortification with graded levels of thymoquinone nanoemulsions (TQN) at 48 and 96 h post-infection with MDR virulent *Pasteurella multocida* strain on relative transcription levels of *P. multocida* virulence genes; *ptfA* **(A)**, *toxA* **(B)** and *nanB* **(C)**. Values are means ± standard error of the mean (SEM) in bars. Control, rabbits fed basal control diets; TQNI, TQNII and TQNIII, rabbits received basal diets with TQN supplementations at graded levels consisting of 100, 200 and 300 mg/kg diets, respectively. ^a–d^Means with various superscript letters denote statistical variations (*p* < 0.05).

The relative transcription levels of *P. multocida* virulence-related genes via RT-qPCR at 48 and 96 h post-infection with MDR virulent *P. multocida* are shown in Figure 3. The outcomes demonstrated that TQN supplementations, especially at higher levels significantly (*p* < 0.05) downregulated the transcription levels of *ptfA, toxA* and *nanB* virulence genes compared to the control non-supplemented group. Of note, TQN dietary supplementation at a concentration of 300 mg/Kg had a noticeable decreasing impact on the expression of the examined virulence genes at 48 and 96 h post-infection with *P. multocida* strain, with a special reference to *nanB* gene (0.41 and 0.33–fold change, respectively).

### 3.8 Gene expression analysis of cytokines-related genes post-infection with *Pasteurella multocida*

**Figure 4:**
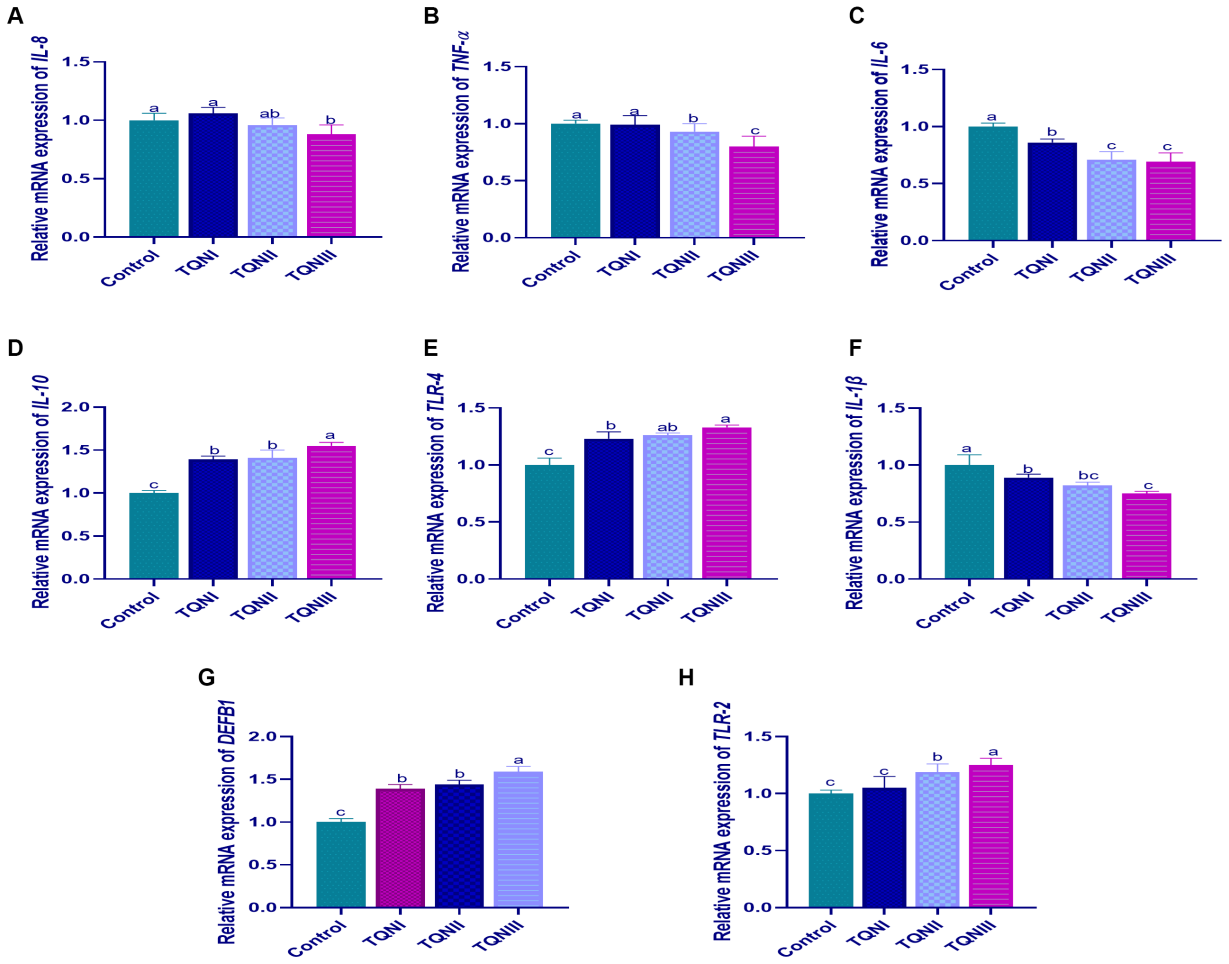
Transcriptional levels of cytokines-related genes; IL-8 [interleukin-8; **(A)**], TNF-α [tumor necrosis factor-alpha; **(B)**], IL-6 [interleukin-6; **(C)**], IL-10 [interleukin-10; **(D)**], TLR-4 [toll-like receptor 4; **(E)**], IL-1β [interleukin-1β; **(F)**], DEFB1 [beta-defensin 1; **(G)**] and TLR-2 [toll-like receptor 2; **(H)**] detectable via RT-qPCR in the splenic tissues of rabbits offered graded levels of thymoquinone nanoemulsions (TQN) supplementations at 96 h post-infection with MDR virulent *Pasteurella multocida* strain. Values are means ± standard error of the mean (SEM) in bars. Control, rabbits fed basal control diets; TQNI, TQNII and TQNIII, rabbits received basal diets with TQN supplementations at graded levels consisting of 100, 200 and 300 mg/kg diets, respectively. ^a–c^Means with various superscript letters denote statistical variations (*p* < 0.05).

The results of mRNA expression levels of cytokines-related genes via RT-qPCR at 96 h post-infection with MDR virulent *P. multocida* strain are illustrated in Figure 4. Our findings showed that supplementing the rabbits with TQN significantly (*p* < 0.05) upregulated the transcription levels of *IL-10* (Figure 4D), *TLR-4* (Figure 4E), *DEFB1* (Figure 4G) and *TLR-2* (Figure 4H) genes comparing with the control non-supplemented rabbits at 96 h post-infection with *P. multocida* strain. Moreover, the results displayed that increasing TQN levels significantly (*p* < 0.05) decreased the expression levels of *IL-8* (Figure 4A), *TNF-α* (Figure 4B), *IL-6* (Figure 4C) and *IL-1β* (Figure 4F) genes un like the control group at 96 h post-infection with *P. multocida*. Of note, rabbits offered 300 mg/kg of TQN had the highest significant (*p* < 0.05) increase in the expression levels of *IL-10*, *TLR-4*, *DEFB1* and *TLR-2* genes (up to 1.59– fold change) and the most significant (*p* < 0.05) downregulation in the transcription levels of *IL-8*, *TNF-α*, *IL-6* and *IL-1β* genes (up to 0.69– fold change) concerning the control group.

## 4 Discussion

Recent developments in global rabbit industry have encouraged the utilization of a variety of practical and cost-effective strategies to recommend the sustainability of rabbit production. Additionally, the excessive usage of antimicrobials led to the development of MDR strains, which limit their use; thus, several researches have focused on finding alternative substances from natural herbs (11, 20). Phytochemicals are thought to have a key role in rabbits’ diets. Among them, TQ was employed as a potential antimicrobial, anti-carcinogenic, antioxidant, anti-inflammatory, immunostimulant and growth-promoting agent (11, 19, 24, 25). Of note, the use of nanotechnology in rabbit breeding remains in its nascent stage and unfortunately, to the best of our knowledge, there are no data on the application of TQN in the rabbit industry. Thus, our study investigated, for the first time, the efficacy of TQN on rabbits’ growth performance, digestive and antioxidant enzymes’ activities, phagocytic percentage, immunological and biochemical markers in addition to *P. multocida* resistance in challenged rabbits.

In the present work, dietary inclusion of TQN had enhancing impacts on rabbits’ growth attributes unlike the control group. Additionally, rabbits receiving TQN at a level of 300 mg/Kg exhibited the maximum BWG and superior FCR throughout the rearing period. In agreement with our outcomes, *Nigella sativa* (NS) seed had growth-enhancing impacts by provoking the BWG and FCR in rabbits (24, 47, 48) and broilers (28). Furthermore, rabbits offered dietary NS extract showed ameliorated FCR and enhanced BWG (11). In broilers, a previous research demonstrated that NS seed had enhancing impact on the digestive system, which led to better growth performance (49). In accordance, previous reports noted that dietary inclusion of TQ had provoked the BWG and FCR in broilers (50) and fish (19). Moreover, a previous report displayed that nano-encapsulated cumin EO had ameliorated the BWG and FCR in broilers (51). However, the effect of TQN on growth performance in rabbits was not studied until now. The high growth performance of rabbits offered TQN supplementation could be attributed to their role in preserving the structure and enhancing the functions of the digestive system as previously recorded (19, 36, 52). The production of digestive enzymes could be altered through hormones, genes and dietary supplements (53). In parallel with the enhanced growth performance in TQN-supplemented groups, the activities of jejunal digestive enzymes (amylase, lipase and trypsin) were likewise raised indicating their improving impact on digestibility and nutrient utilization. In agreement with our outcomes, a recent study reported that NS extract had improving impacts on rabbits’ growth, feed utilization, digestive enzymes and gut microbial flora (11). A recent report revealed also that TQ enhanced digestion (54) and this may be linked to its function as a potent stimulator of digestive enzymes and its ability to increase nutrient retention with a favorable impact on nutrient utilization and growth performance (55). Previous studies stated the enhancing effects on broilers’ digestive enzymes following dietary fortification with eugenol nanoemulsions (56), garlic nano-hydrogel (57), thymol nanoemulsion (31) and EOs mixture (18). However, the effect of TQN on the activities of digestive enzymes in rabbits has not yet been investigated. Of note, EOs may affect growth rate by increasing nutrient digestibility through the regulation of the intestinal microbiota and upregulation of endogenous enzymes (58). Additionally, it was formerly proven that phytochemicals can modify the digestive transcriptional patterns (59). The differences in the impacts of EOs between various researches may be resulted from the variability in supplementation doses, components, extraction techniques and sources (19). The enhancing effect of thymoquinone nanoemulsion on rabbits’ performance might be explained by improving the bioactivity and bioavailability of TQ because TQN permit easier uptake by cells in the gut and deeper penetration to tissues resulting in an effective rise in the transcription levels of genes encoding digestive enzymes (31).

The immune system and overall health of animals are closely related to the antioxidant defense system. Subjecting animals to stressful situations could increase the formation of reactive oxygen species (ROS), which cause oxidative stress and significant cell damage. The antioxidant defense system helps the animals to keep endogenous ROS at shallow concentrations and reduce oxidative damage brought on by the elevated reactive nature of ROS (60). Due to the ongoing formation and clarity of free radicals by the animal’s antioxidant system, their levels are maintained under a dynamic balance in normal physiological conditions (61). On the other hand, increasing ROS generation might promote the lipid peroxidation of cell membranes and have a detrimental effect on animal health and performance (62). Antioxidant enzymes, including GPX, SOD and CAT, are thought to be the primary lines of defense against the production of harmful ROS, resulting in direct detoxification (63, 64). The T-AOC is regarded as an index to reflect the body’s antioxidant levels (65). Contrarily, larger levels of free radicals lead to an excess of MDA, which is one of the byproducts of cells’ lipid peroxidation; thus, the MDA concentration is typically used as a sign of oxidative stress (66). Animal diets supplemented with antioxidants can eliminate free radicals, which can coordinate the animal’s antioxidant system. In this situation, TQN is a natural antioxidant, but researches on the processes by which it affects the rabbits’ antioxidant system and whether its application would offer an extra advantage for enhancing this function are currently lacking and require more investigation. Our results showed a significant increase in the concentrations of T-AOC and GPX, CAT and SOD enzymes and a decrease in the level of MDA in the breast muscle and jejunal tissues of rabbits offered dietary TQN in comparison with the control group suggesting its role in activating the antioxidant enzymatic processes. Similarly, TQ dietary supplementation increased the activities of CAT, SOD and GPX antioxidant enzymes and lowered the concentrations of MDA in rabbits (67). In accordance, dietary fortifications with liposomal encapsulated TQ elevated the levels of T-AOC, CAT antioxidant enzyme and decreased the level of MDA in rabbits (68). Furthermore, dietary inclusion of microalgae mixture increased the activities of T-AOC and GPX, CAT and SOD antioxidant enzymes and decreased the level of MDA in fish, which enhanced the cells’ resistance to oxidative stress (69). Numerous studies have stated that TQ has antioxidant properties, which are responsible for eliminating free radicals and provide a significant improvement to oxidative stress responses (19, 70, 71). Herein, dietary inclusion of TQN boosted the antioxidant potential of rabbits, which might be attributed to the effectiveness of nanoemulsions in enhancing the bioactivity and bioavailability of TQ, which allow easier uptake by cells and deeper penetration to tissues leading to enhancement of antioxidant defense system.

The liver enzymes aminotransferases (AST and ALT) reflect the health condition of the liver (39). Herein, TQN supplementations did not change the levels of AST and ALT, which suggested that TQN has a protective impact on the rabbits’ liver tissues. In agreement with our outcomes, dietary inclusion with liposomal encapsulated curcumin and nano-curcumin did not alter the activities of AST and ALT (39, 72). Similarly, previous studies stated that dietary inclusion of EOs did not alter the levels of AST and ALT in fish (19) and broilers (40). Furthermore, dietary EOs (73) and coconut oil (74) fortifications reduced the serum level of ALT and AST in rabbits, respectively. Our findings displayed a significant decrease in the activities of total cholesterol, TG and LDL in rabbits receiving dietary TQN, which suggests that TQN might have a modulatory effect in regulating the level of enzymes involved in lipid metabolism. Moreover, lowered serum cholesterol concentrations were also reflected in reduced cholesterol levels in thigh and breast muscles after TQN dietary supplementations. Our findings might be attributed to boosting the cholesterol total fecal excretion through the bile and inhibiting its absorption in the gut (40, 75). Additionally, phytochemical nanoemulsions may alter hepatic gene expressions and prevent the biosynthesis of cholesterol by decreasing important lipogenic factors that promote the production of bile acids and boost cholesterol clearance (76). Moreover, TQ dietary supplementation minimized the serum levels of TC and TG in fish (19). In accordance, EOs supplementations decreased the serum activities of TC, TG and LDL in broilers (40). Accordingly, similar outcomes were observed when abdominal fat, cholesterol levels in the thigh and breast muscles and serum levels of TC, TG and LDL were reduced in broilers receiving dietary NS supplementations (77).

The health of animals is primarily maintained by the immune system. Essential oils have a favorable impact on the animals’ immune defense as they improve the activities of lymphocyte and immunoglobulin synthesis (78, 79). The rabbits’ immunological response and antioxidant defense systems are positively correlated providing defense against invasive harmful microorganisms. Strengthening the immune defense of rabbits through dietary natural antioxidants could counteract the issue of stressful situations during the weaning phase. Total Igs have significant functions in the immunological activities including phagocytosis and neutralization of harmful microorganisms making them important components of the humoral immune defense (80). Additionally, IgM, IgG and IgA are the three main immunoglobulin isotypes that react to both systemic and local microorganisms (81). Our outcomes showed that rabbits offered dietary TQN, particularly at a level of 300 mg/kg showed increases in the levels of IgG, IgM, IgA and total Igs and phagocytic percentage when compared with the control group. These findings were attributed to provoking the rabbits’ immune system, which accelerated the synthesis of cytokines that are essential for controlling the immune response (76). Similarly, dietary coconut oil supplementation enhanced the phagocytic activity and immune response in rabbits (74) and dietary inclusion with NS seed enhanced the serum levels of IgG and IgM in rabbits (24). Moreover, TQ dietary supplementation enhanced the serum level of IgM in fish (19). Furthermore, EOs fortifications provoked the serum levels of IgG and IgM and blood phagocytic percentage in rabbits (74). In accordance, a recent study displayed that phytochemical curcumin encapsulation increased the levels of total Igs and IgM in fish (39). Accordingly, previous reports displayed that EOs supplementation enhanced the activities of IgG, IgM and IgA and phagocytic percentage in broilers (40) and piglets (82).

*Pasteurella multocida* can colonize the respiratory tract of rabbits causing snuffles (pasteurellosis) and significant financial losses for rabbit breeding (5, 6). For many years, antimicrobials have been considered a key tool in the treatment of bacterial infections; but as a result of their excessive usage, MDR strains have emerged recently (21). From this perspective, the EOs’ possible benefits have been considered as a possible approach to combat bacterial infections (18, 19, 22). There is a lack of information regarding EOs’ ability to prevent or treat *P. multocida* infections in farmed rabbits. Herein, quantitative analysis of *P. multocida* counts in challenged rabbits displayed that dietary inclusion with TQN significantly minimized *P. multocida* loads at 48 and 96 h post-infection with MDR virulent *P. multocida* strain concerning the control group. Similarly, a recent study reported that NS extract significantly minimized methicillin-resistant *Staphylococcus aureus* counts in challenged rabbits suggesting its antibacterial activities (11); but, the impact of TQN on *P. multocida* populations in the lung tissues of challenged rabbits was not studied till now. Regarding *P. multocida* resistance in challenged rabbits following TQN fortifications, previous studies stated that NS extract can alter the innate immune response function by minimizing bacterial survival, raising nitric oxide generation and improving the phagocytic ability of macrophages (11, 83).

Nutritional immunology is a unique approach to disease prevention in the rabbit industry via the use of dietary supplements to get around the limitations of vaccination programs (11, 84). Moreover, improving rabbits’ nutrition and veterinary care for preventing infections will increase the economic and productive efficiency of rabbits’ husbandry. Notably, numerous treatment strategies target recently microbial pathogenicity rather than microbial survival (85). Thus, the TQN anti-virulence activities were investigated by determining the transcription levels of *ptfA, toxA* and *nanB* virulence genes in response to its supplementation at 48 h and 96 h post-infection with *P. multocida* strain. Herein, the transcription levels of *ptfA, toxA* and *nanB* virulence genes were significantly downregulated in challenged rabbits fortified with TQN supplementation, especially at high levels, unlike the control group at 48 h and 96 h post-infection with MDR virulent *P. multocida* strain. Similarly, a previous report displayed the *in vivo* anti-virulence characteristics of dietary TQ supplementations *against Aeromonas sobria* in challenged fish (19). Furthermore, a recent study stated the *in vivo* anti-virulence properties of thymol nanoemulsion against *Salmonella Enteritidis* in challenged broilers (30). Notably, a recent study displayed that marjoram extract downregulated the expressions of *P. multocida* virulence-related genes *in vitro* (6); however, the *in vivo* anti-virulence effect of TQN in rabbits experimentally infected with *P. multocida* has not yet been studied. The anti-virulence characteristics of TQN might be resulted from the inhibition of quorum sensing (QS), which is the microbial gene regulation system that controls the transcription of various virulence markers (19). A recent report stated that the EO suppresses QS at sub-inhibitory concentrations and reduces a range of QS markers in a dose-dependent effect (86). The direct impact of EO on the production of QS signaling molecules and the deactivation of cognate receptors may be responsible for its anti-QS effects. As a result, it inhibited the transcription of virulence genes responsible for cooperative behaviors (30, 85).

The persistence of gut integrity and barrier functions can be influenced by the interactive effects of phytochemicals on the superior immune response via altering the transcription of various cytokines, mucin and pattern recognition receptors (87). It is well-recognized that cytokines have a significant regulatory role in controlling the gut inflammatory reactions (84). Notably, the tight junctions serve as both physical and functional barriers against the entry of harmful pathogens and other chemicals making them essential elements of the gut barrier functions (88). Numerous markers such as mucin, tight junction molecules and defensins reflect different elements of the gut barrier. Moreover, TLR signaling improves the integrity of tight junctions by improving the transcription of important genes related to tight junctions (11). Of note, TQ has immunostimulant activities in a variety of animals’ immunologic and inflammatory illnesses (89) and it exerts an ameliorating impact on the anti-inflammatory cytokines such as *IL-10* and the proinflammatory cytokines including *TNF-α*, *IL-8*, *IL-6* and *IL-1β*, which inhibits the development of intestinal inflammation and maintains intestinal hemostasis (90). Contrarily, when bacteria invade the gut epithelial cells, gut immune cells begin cytokines synthesis, which in turn promotes immune defense against bacteria (91). In this context, *P. multocida* may increase the expression levels of *TNF-α*, *IL-8* and *IL-6* genes, which in turn raises the gastrointestinal epithelium’s permeability (92). *IL-1β* is one of the most important pro-inflammatory cytokines, which promotes its own expression as well as the transcription of other chemokines and pro-inflammatory cytokines leading to recruiting the inflammatory reactions and initiating the formation of antimicrobial cells (93). Additionally, *IL-10* has a primarily antagonistic effect on inflammation in addition to its critical function in suppressing immunological and inflammatory reactions (94). Our findings showed that, in parallel with improving the serum immunological parameters and phagocytic indices, dietary TQN fortifications significantly downregulated the expression levels of *TNF-α*, *IL-8*, *IL-6* and *IL-1β* genes and upregulated the transcription levels of *IL-10*, *TLR-4*, *DEFB1* and *TLR-2* genes in rabbits at 96 h post-infection with *P. multocida* in comparison with the control group. Our findings indicate that TQN fortifications successfully counteracted the strong inflammatory reactions in *P. multocida* experimentally-infected groups suggesting its potent anti-inflammatory and immunostimulant activities. In agreement with our findings, TQ can control the movement of inflammatory cells by altering the transcription of cytokines and/or chemokines, which has the effect of reducing the immune system’s response to inflammation (89). In a comparable study, NS extract reduced the expression levels of *TNF-α*, *IL-8*, *IL-6* and *IL-1β* genes and increased the transcription levels of *IL-10*, *TLR-4*, *DEFB1* and *TLR-2* genes in rabbits (11) and dietary supplementations of NS powder improved the immunological responses in broilers (95). These findings may provide an explanation for TQ anti-inflammatory properties (90). Thymoquinone has already been shown to have improving effects on the immune defense via enhancing the response of antibodies and restoring the immunological and inflammatory changes (96). From our point of view, TQN have antimicrobial, antioxidant, immunostimulant and anti-inflammatory properties that enhance serum cellular and humoral immunity, which in turn reduces the proliferation of harmful microorganisms and inflammation in rabbits.

## 5 Conclusion

Overall, the outcomes of our work indicated the improving effects of TQN dietary supplementation during the entire experimental period on rabbits’ growth, digestion, immunity and antioxidant potential as realized by increasing the levels of digestive and antioxidant enzymes as well as biochemical and immunological markers. Moreover, dietary inclusion of TQN for rabbits challenged with *P. multocida* reduced the severity of clinical manifestations and bacterial translocation or localization by reducing the counts of *P. multocida* in rabbits’ lungs, downregulating the expression levels of *P. multocida* virulence-related genes and ameliorating the transcription levels of cytokines-related genes. Therefore, our findings suggest using TQN as a novel dietary supplement, which is advertised to play a fundamental role in controlling *P. multocida* infection in rabbits.

## Data availability statement

The original contributions presented in the study are included in the article/supplementary material, further inquiries can be directed to the corresponding authors.

## Ethics statement

The animal study was approved by all procedures for experiments were performed following the rules and authorized specifications of the Institutional Animal Care and Use Committee (IACUC), Faculty of Veterinary Medicine, Zagazig University, Egypt under the reference number (ZU-IACUC/2/F/195/2022). The study was conducted in accordance with the local legislation and institutional requirements.

## Author contributions

MA: Data curation, Investigation, Methodology, Resources, Writing – original draft, Writing – review & editing. ME-A: Investigation, Writing – original draft. RE-M: Methodology, Software, Validation, Writing – review & editing. ME: Formal analysis, Funding acquisition, Resources, Software, Visualization, Investigation, Writing – original draft. AA: Supervision, Writing – original draft, Writing – review & editing. EY: Conceptualization, Investigation, Visualization, Writing – original draft. WY: Data curation, Investigation, Writing – original draft, Writing – review & editing. RD: Conceptualization, Data curation, Formal analysis, Funding acquisition, Writing – original draft. DE: Investigation, Methodology, Project administration, Writing – review & editing. MH: Software, Supervision, Validation, Visualization, Writing – review & editing. AO: Data curation, Funding acquisition, Resources, Visualization, Writing – review & editing. SM: Methodology, Resources, Visualization, Writing – original draft. SD: Writing – original draft. DI: Conceptualization, Formal analysis, Methodology, Supervision, Writing – original draft, Writing – review & editing.
