# Corrigendum: Future impact of thymoquinone-loaded nanoemulsion in rabbits: prospects for enhancing growth, immunity, antioxidant potential and resistance against *Pasteurella multocida*

**DOI:** 10.3389/fvets.2024.1388859

**Published:** 2024-03-01

**Authors:** Marwa I. Abd El-Hamid, Mona M. El-Azzouny, Rania M. S. El-Malt, Mona E. Elkenawy, Abdelwahab A. Abdelwarith, Elsayed M. Younis, Wessam Youssef, Rehab E. Dawod, Dalia W. A. H. Elged, Manal A. M. Habaka, Amal S. A. El Oksh, Soad Mekawy, Simon J. Davies, Doaa Ibrahim

**Affiliations:** ^1^Department of Microbiology, Faculty of Veterinary Medicine, Zagazig University, Zagazig, Egypt; ^2^Department of Bacteriology, Animal Health Research Institute (AHRI), Agriculture Research Center (ARC), Zagazig, Egypt; ^3^Department of Biochemistry, Animal Health Research Institute (AHRI), Agriculture Research Center (ARC), Mansoura, Egypt; ^4^Department of Zoology, College of Science, King Saudi University, Riyadh, Saudi Arabia; ^5^Department of Biotechnology, Animal Health Research Institute (AHRI), Agriculture Research Center (ARC), Giza, Egypt; ^6^Department of Bacteriology, Animal Health Research Institute (AHRI), Agriculture Research Center (ARC), Damietta, Egypt; ^7^Toxicology and Biochemical Department, Animal Health Research Institute (AHRI), Agriculture Research Center (ARC), Zagazig, Egypt; ^8^Department of Poultry and Rabbits Diseases, Animal Health Research Institute (AHRI), Agriculture Research Center (ARC), Zagazig, Egypt; ^9^Department of Biotechnology, Reference Laboratory for Quality Control of Poultry Production (RLQP), Animal Health Research Institute (AHRI), Agriculture Research Center (ARC), Zagazig, Egypt; ^10^Department of Clinical Pathology, Animal Health Research Institute (AHRI), Agriculture Research Center (ARC), Zagazig, Egypt; ^11^Aquaculture Nutrition Research Unit (ANRU), Carna Research Station, College of Science and Engineering, Ryan Institute, University of Galway, Galway, Ireland; ^12^Department of Nutrition and Clinical Nutrition, Faculty of Veterinary Medicine, Zagazig University, Zagazig, Egypt

**Keywords:** thymoquinone nanoemulsions, growth, immunostimulant, antioxidant, anti-virulence, *Pasteurella multocida*

In the published article, there was an error in the Funding statement. The funding project number was mistakenly written as RSPD2024R700 instead of RSP2024R36. The correct Funding statement appears below.

